# Ischemic Stroke of the Corpus Callosum Mimicking Cerebellar-Type Ataxia: A Case Report and Diagnostic Pitfall

**DOI:** 10.7759/cureus.114493

**Published:** 2026-08-13

**Authors:** Nisrine El Guerouani, Wadii Bnouhanna, Mounia Rahmani, Maria Benabdeljlil, Saadia Aïdi

**Affiliations:** 1 Department of Neurology A and Neuropsychology, Specialties Hospital of Rabat, Avicenne University Hospital, Rabat, MAR; 2 Department of Neurology A and Neuropsychology, University Mohammed V, Rabat, MAR

**Keywords:** ataxia, corpus callosum, dysarthria, esus, interhemispheric disconnection, splenium, stroke, toast criteria, vascular disease

## Abstract

Infarction of the corpus callosum (CC) remains relatively rare, largely because of its extensive vascular supply from both the anterior and posterior cerebral circulations. When this occurs, the clinical presentation generally consists of nonspecific motor weakness or language disturbances. Ataxia is highly atypical in this context. We present the case of a 65-year-old diabetic woman who presented with acute-onset cerebellar ataxia, dysarthria, recent memory impairment, disorientation, and a few subtle signs of interhemispheric disconnection. Brain imaging showed an acute ischemic lesion involving the body and splenium of the CC along with chronic extra-callosal infarcts. The patient showed a favorable clinical outcome following antiplatelet therapy and secondary vascular prevention. The mechanisms underlying the ataxia remain uncertain. Through this case, we aim to highlight the key clinical, radiological, and etiological features of callosal infarction and the challenges of attributing its manifestations to a single anatomical mechanism.

## Introduction

The corpus callosum (CC), the largest interhemispheric commissure, is particularly well vascularized by both the anterior and posterior cerebral circulations, which anastomose at the level of the splenium [1,2]. It is involved in transferring information between the cerebral hemispheres [1]. Indeed, this dual vascularization explains why callosal infarction remains a relatively rare event [3], representing only 3.6% of all ischemic strokes [2]. In the same cohort, isolated callosal infarction accounted for only 0.4% of ischemic strokes [2].

Callosal infarction is characterized by a complex and nonspecific clinical picture, which is often responsible for delayed or missed diagnoses, especially in cases of associated lesions. The classical callosal disconnection syndrome, which comprises cortical dysfunctions such as apraxia, agraphia, tactile anomia, and alien hand syndrome, occurs predominantly when the CC is severely damaged [4,5]. Ataxia occurs in approximately 10% of cases [2], which is an uncommon clinical manifestation given the lack of direct connections with the cerebellum. The association with extra-callosal lesions can complicate clinical localization. The objective of this report is to describe an atypical presentation of callosal infarction, focusing on its clinical, radiological, and etiological features.

## Case presentation

A 65-year-old woman with insulin-treated type 2 diabetes and no relevant neurological history, independent in basic and instrumental activities of daily living (mRS 0), experienced sudden-onset gait instability that was maximal at symptom onset (Day 0). Two days later (Day 2), she developed dysarthria, prompting her to seek medical attention at the emergency department. She was subsequently admitted to our neurology department four days after the initial onset of symptoms (Day 4).

On admission, neurological examination revealed marked gait ataxia and dysarthria, with prominent truncal incoordination. No motor or sensory deficits were observed. Deep tendon reflexes were present and symmetric. Proprioception was preserved. The Romberg test could not be performed because the patient was unable to maintain an upright standing position without assistance. Cranial nerve examination was normal, with no nystagmus. Finger-to-nose and heel-to-knee testing were normal, without dysmetria or decomposition. Cognitive screening using the Moroccan-adapted version of the Montreal Cognitive Assessment yielded a score of 8/30, indicating marked cognitive impairment, particularly affecting memory, attention, and orientation [6]. The patient was illiterate, and her educational background was taken into consideration when interpreting the score. Assessment of praxis and tactile gnosis disclosed a mild apraxic disturbance and tactile anomia, without clear lateralization to either upper limb. The National Institutes of Health Stroke Scale score was 3 on admission. Given the predominant posterior circulation features, it was considered insufficient to fully reflect the clinical severity.

Regarding vascular risk factors, she had no history of hypertension but was obese. Laboratory investigations showed poor glycemic control and an abnormal lipid profile (Table 1).

**Table 1:** Laboratory parameters HbA1c: hemoglobin A1C, LDL: low-density lipoprotein, HDL: high-density lipoprotein

Parameter	Results	Reference range
Fasting blood glucose	1.44 g/L	<1.26 g/L
HbA1c	7.7%	<6%
Total cholesterol	1.99 g/L	<2 g/L
LDL‑cholesterol	1.27 g/L	<1.6 g/L
HDL‑cholesterol	0.28 g/L	0.4-0.6 g/L
Triglycerides	2.19 g/L	<1.5 g/L

**Figure 1:**
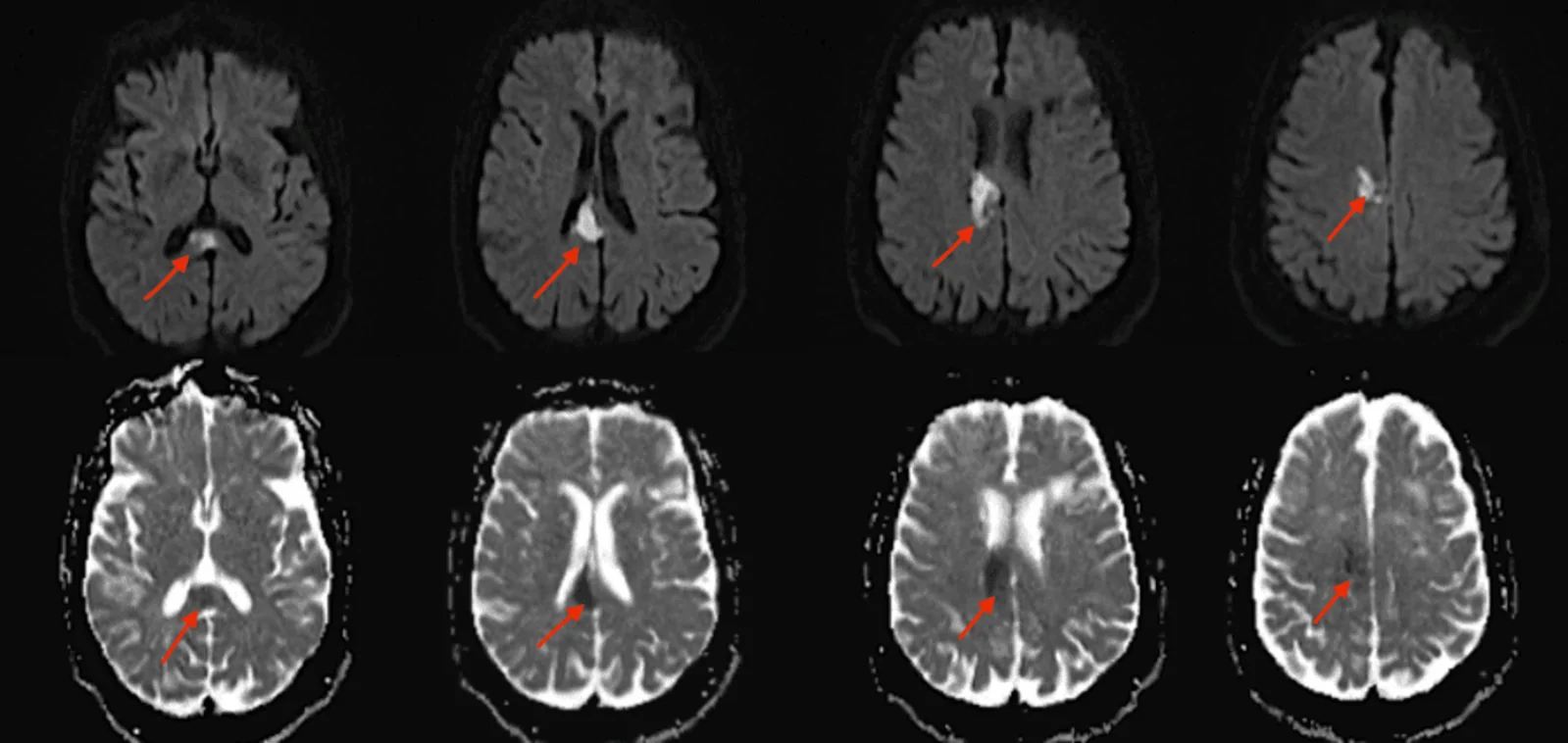
Acute callosal ischemic stroke on brain MRI axial DWI showing a hyperintense signal (red arrows, top row) with restricted diffusion on the ADC map (red arrows, bottom row), involving the posterior two-thirds of the body and the splenium of the CC MRI: magnetic resonance imaging, DWI: diffusion-weighted imaging, ADC: apparent diffusion coefficient, CC: corpus callosum

**Figure 2:**
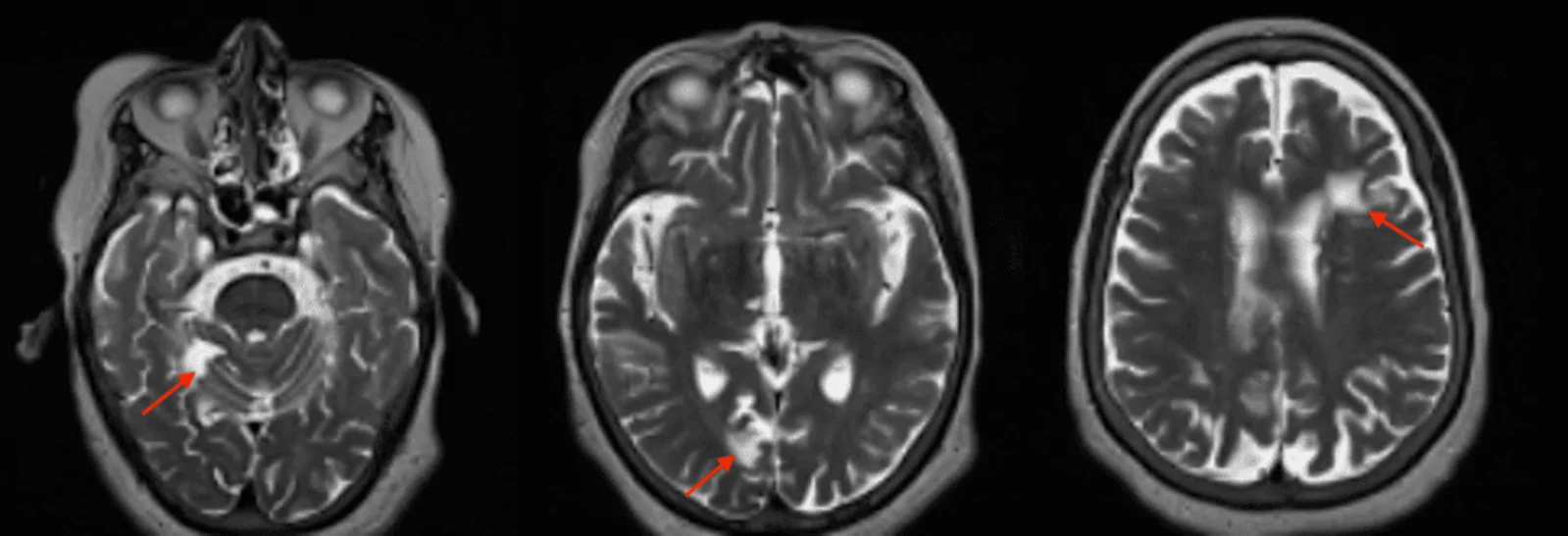
Axial T2‑weighted MRI showing multiple hyperintense lesions (red arrows) in the left frontal, right cerebellar, and right occipital regions. These lesions were consistent with chronic ischemic changes on the basis of the full MRI sequences (FLAIR, T1‑weighted, and DWI) MRI: magnetic resonance imaging, FLAIR: fluid-attenuated inversion recovery, DWI: diffusion-weighted imaging

**Figure 3:**
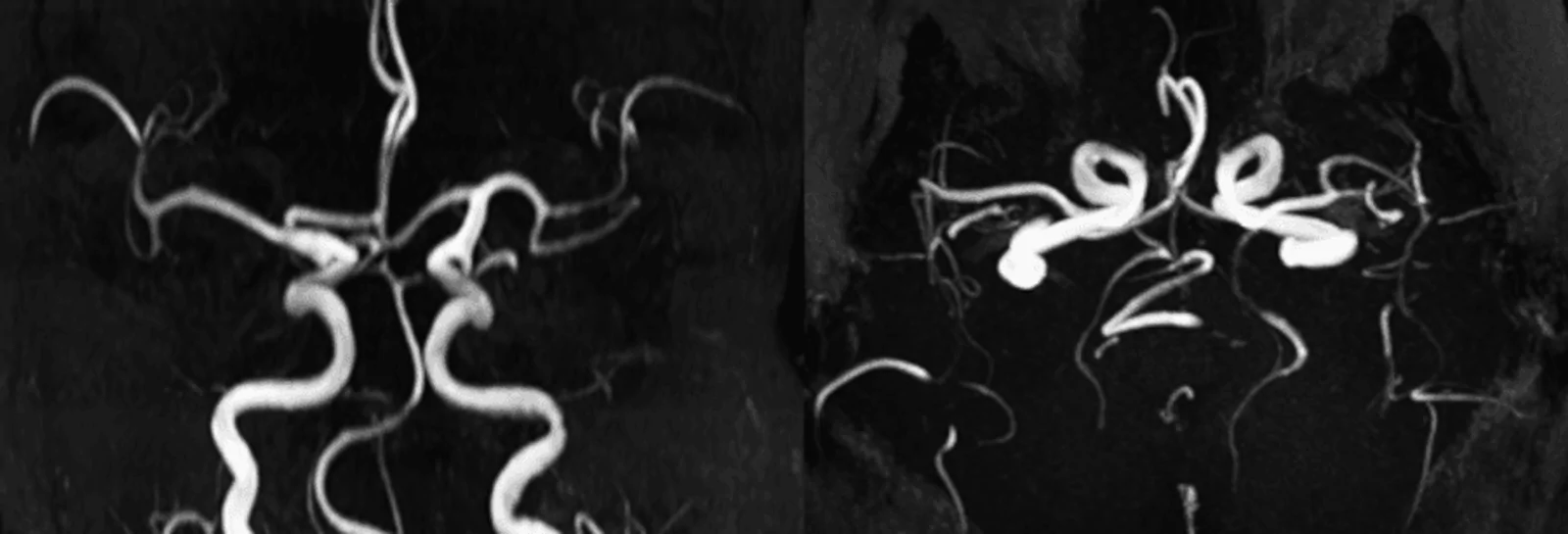
MR angiography of the circle of Willis time-of-flight sequence showing no arterial abnormality and no significant intracranial arterial stenosis or occlusion MR: magnetic resonance

Brain MRI confirmed the diagnosis by demonstrating a hyperintense lesion on diffusion-weighted imaging (DWI) with corresponding restricted diffusion on the apparent diffusion coefficient (ADC) map (Figure 1). The infarction involved the posterior two-thirds of the body and the splenium of the CC, indicative of acute callosal infarction. Multiple chronic ischemic lesions (left frontal and right occipital and cerebellar) were also identified (Figure 2). Magnetic resonance angiography of the circle of Willis and Doppler ultrasonography of the supra-aortic trunks showed no significant abnormalities (Figure 3).

A complete cardiovascular evaluation, including electrocardiography and transthoracic echocardiography, revealed no abnormalities. A 24-hour Holter ECG was also performed but failed to identify any cardiac arrhythmia. Given the involvement of multiple vascular territories, an embolic mechanism was considered; however, no cardiac source was identified. According to the TOAST classification, the stroke was therefore classified as of undetermined etiology, with a possible embolic mechanism. The patient was managed with antiplatelet therapy (160 mg per day of aspirin), cardiovascular risk-factor control (20 mg per day of rosuvastatin), strict blood pressure monitoring, adjustment of insulin doses to optimize glycemic control, and early rehabilitation. At discharge, after a 16-day hospital stay, the patient showed marked improvement in gait ataxia. The patient was lost to follow-up and did not attend any further scheduled visits.

## Discussion

Two reasons explain the rare occurrence of callosal infarctions. First, the CC receives a particularly rich, dual blood supply from both the anterior and posterior circulations. The anterior circulation contributes through the pericallosal artery, a branch of the anterior cerebral artery, as well as the subcallosal and median callosal arteries, which arise from the anterior communicating artery. The posterior circulation is supplemented by the posterior pericallosal artery, a branch of the posterior cerebral artery. Additionally, the perforating arteries of the CC originate perpendicularly from their trunks, providing an anatomical configuration that protects against embolic occlusion [1-4]. Second, the CC is also a densely packed white matter fiber tract, making it relatively resistant to hypoxia or transient ischemia compared to gray matter [2,4].

For these reasons, isolated CC infarction is highly uncommon. Callosal infarction more often occurs in association with hemispheric lesions; in our patient, multiple chronic extracallosal ischemic lesions were also identified. The splenium is the most commonly involved region, possibly because of its location in an area of overlap between the anterior and posterior cerebral circulations, with many infarctions involving the territory of the posterior cerebral artery [3]. In our patient, involvement was not limited to a single region; the lesion extended to the body and splenium, possibly reflecting poor collateralization between the anterior and posterior circulations [2,4].

Ataxia is not a common manifestation of callosal infarction. In the largest series, Li et al. reported ataxia in only six out of 59 patients (10.1%), placing it among the least frequent neurological signs, significantly less common than hemiparesis (72.9%), sensory abnormalities (32.2%), and dysarthria (22.0%) [2]. What makes this observation interesting is that, in our case, ataxia and dysarthria were the main complaints, which initially led to suspicion of a cerebellar lesion. Brain imaging subsequently revealed a CC lesion, highlighting the contrast between the initial clinical presentation and the lesion location. Such a diagnostic pitfall is precisely why our case is worth reporting. The patient presented for medical evaluation four days after symptom onset. This delay may explain the partial regression of symptoms and, consequently, the less complete clinical picture at the time of neurological examination.

However, ataxia has no localizing value within the CC, as it occurs with nearly equal frequency in infarcts of the anterior genu and/or body (13.0%) and infarcts of the posterior splenium (11.5%) [2]. This suggests that the presence of ataxia relates more to involvement of associated cerebellar or sensory pathways than to the callosal lesion itself. In the case of our patient, the mechanism of this ataxia deserves particular attention, since there was no acute cerebellar involvement. The cerebellar lesion was chronic and had been asymptomatic prior to this episode; otherwise, it would have been a competing localization to explain the ataxia. The most plausible explanation would be the interruption of association fibers passing through the CC, resulting in a disconnection syndrome. The generally favorable short-term prognosis of isolated CC infarction may be related to its abundant blood supply. Zhang et al. also proposed that the CC may predominantly undergo demyelination rather than neuronal degeneration or necrosis after infarction, with subsequent remyelination potentially contributing to recovery [4].

The classical disconnection syndrome is suggestive of diffuse or bilateral CC involvement but is exceptionally complete [5]. Our patient had only partial manifestations: a minimal apraxic disturbance and a mild, non-lateralized tactile anomia, with no evidence of alien hand phenomena. The cognitive impairment may be related, at least in part, to disruption of commissural fibers connecting association areas across the two hemispheres. However, the precise contribution of interhemispheric disconnection remains speculative and cannot be established in a single case. This impairment may have been further influenced by the pre-existing frontal infarct, which may have affected executive function; however, no premorbid cognitive assessment was available [5]. Ischemic lesions involving the entire CC have also been described in association with a clinical picture of dementia [7].

Diffusion-weighted MRI remains the imaging modality of choice for confirming the diagnosis. As the most sensitive sequence in the acute phase, DWI shows lesion hyperintensity with a corresponding decrease in ADC values, reflecting cytotoxic edema [5,7]. In addition to its sensitivity, DWI is valuable for differentiating ischemic lesions from other pathologies that involve the CC. Although ischemia is the most common vascular cause of CC lesions, less frequent vascular causes include vasospasm, venous thrombosis, vasculitis, hypercoagulable states, and global hypoxia. Additionally, non-vascular conditions such as demyelinating diseases, metabolic disorders, and infectious processes may also involve the CC. They should be carefully considered according to the clinical context and radiological pattern [7,8].

Etiologically, atherosclerosis remains the main cause of callosal infarction, while embolism is more frequently implicated when the splenium is affected [4]. The vascular risk factors observed are similar to those of strokes in other cerebral territories. Systemic hypertension is the most frequently reported risk factor, observed in 72.0-73.3% of cases, followed by dyslipidemia and diabetes mellitus, which are both present in our patient, and smoking [3,4,9].

In our patient, the presence of infarcts in both the carotid and vertebrobasilar territories suggested a possible embolic mechanism, particularly in the absence of any detectable abnormality on vascular imaging. This prompted us to pursue a complete cardiovascular investigation, including electrocardiography, transthoracic echocardiography, and 24‑hour Holter ECG monitoring, all of which remained negative and non‑contributory. At this stage, the stroke was classified as being of undetermined etiology according to the TOAST classification. It also fulfilled the criteria for an embolic stroke of undetermined source.

Finally, the six‑month functional outcome reported by Zhang et al. [4] in patients with isolated CC infarction was more favorable than that observed in patients with other infarction types, as was the case in our patient. This favorable outcome has been speculated to be due to the rich collateral vascularization of the CC and predominant demyelination rather than irreversible neuronal necrosis, which may facilitate rapid recovery. However, in the same cohort, diabetes mellitus, multiple cerebrovascular stenoses, and diffuse or extensive infarction were associated with poorer functional outcomes [4].

## Conclusions

Callosal infarction is an uncommon ischemic stroke subtype with subtle and often nonspecific clinical features, making early diagnosis challenging. Our case illustrates this diagnostic difficulty, as the clinical presentation initially suggested a cerebellar lesion until MRI confirmed a callosal infarction. The key diagnostic message is that CC infarction can present with cerebellar‑type ataxia and should be considered when MRI shows an atypical lesion distribution. A standard etiologic workup for ischemic stroke is therefore warranted in such cases. Regarding prognosis, our patient showed favorable short‑term recovery, although outcomes are variable and depend on lesion extent, associated infarcts, and comorbidities.
